# Acoustic indices provide information on the status of coral reefs: an example from Moorea Island in the South Pacific

**DOI:** 10.1038/srep33326

**Published:** 2016-09-15

**Authors:** Frédéric Bertucci, Eric Parmentier, Gaël Lecellier, Anthony D. Hawkins, David Lecchini

**Affiliations:** 1 USR 3278 CNRS-EPHE-UPVD, Paris Sciences et Lettres (PSL), CRIOBE, Moorea, French Polynesia; 2 Laboratoire de Morphologie Fonctionnelle et Evolutive, University of Liège, Liège, Belgium; 3 Université de Versailles, Saint Quentin en Yvelines, France; 4 Laboratoire d’Excellence “CORAIL”, Moorea, French Polynesia; 5 Loughine Marine Research, Aberdeen, UK

## Abstract

Different marine habitats are characterised by different soundscapes. How or which differences may be representative of the habitat characteristics and/or community structure remains however to be explored. A growing project in passive acoustics is to find a way to use soundscapes to have information on the habitat and on its changes. In this study we have successfully tested the potential of two acoustic indices, i.e. the average sound pressure level and the acoustic complexity index based on the frequency spectrum. Inside and outside marine protected areas of Moorea Island (French Polynesia), sound pressure level was positively correlated with the characteristics of the substratum and acoustic complexity was positively correlated with fish diversity. It clearly shows soundscape can be used to evaluate the acoustic features of marine protected areas, which presented a significantly higher ambient sound pressure level and were more acoustically complex than non-protected areas. This study further emphasizes the importance of acoustics as a tool in the monitoring of marine environments and in the elaboration and management of future conservation plans.

Coral reef ecosystems are among the most biologically diverse and complex marine ecosystems worldwide. In addition to their biological and ecological importance, coral reefs support major economic and physical functions (e.g. food production, tourism, biotechnology development and coastal protection) that are essential for many countries^1^. Unfortunately, coral reefs are severely threatened: 20% of coral reefs can no longer be defined as such, another 25% are currently endangered and another 25% will be endangered by 2050 2. The frequency and severity of natural perturbations (e.g. cyclone, outbreaks of predators, particularly the crown-of-thorns starfish [COTS] *Acanthaster planci*) and anthropogenic perturbations (e.g. ocean acidification, pesticides, rising sea-water temperatures) on coral reefs have greatly increased worldwide in the last three decades, and, as a consequence, reef communities (fish, coral and benthic invertebrates) have suffered unprecedented levels of decline^3^,^4^. In this context of increasing degradation of coral reefs, ecological surveys are needed to document changes in reef communities (i.e. “monitoring”) and the impacts of natural and anthropogenic disturbances^4^,^5^,^6^.

While monitoring requires visual observations and the counting of organisms^3^,^6^, recent advances have shown that the collection of acoustical data can complement these efforts. The term ‘soundscape’ describes the physical combination of sounds that prevails at a particular place and time^7^. A number of acoustic indices, providing single, easy to interpret values, have been developed to assess the complexity and dynamics of soundscapes^8^,^9^. For example, acoustic indices have revealed lower acoustic diversity in disturbed Tanzanian forests, with altered community composition, compared to preserved forests^10^. Towsey *et al*.^11^ showed that the use of 14 different bioacoustic indices significantly improved evaluation of the bird diversity. Soundscape analysis can provide a valuable tool for studying and monitoring animal diversity, abundance, behaviour, dynamics and the distribution of calling animals in the terrestrial environment^12^.

The ocean has never been a silent world. Ambient sea noise is composed of sounds generated by abiotic sources such as wind, waves and ground movement (geophony) and biotic sounds (biophony) produced by various marine organisms^13^. A third source of sounds produced through human activities (anthropophony) is now common, especially in coastal marine environments^14^,^15^. Together, geophony, biophony and anthropophony combine to create the soundscape of a particular environment^8^. Several recent studies have shown that marine larvae, which have specialised habitat requirements, may use sounds to locate and orientate towards their habitats when returning to the reef^16^,^17^, explaining the differential patterns of settlement within heterogeneous environments^18^,^19^. Additionally, in recent years, passive acoustics monitoring has allowed biotic activity to be monitored remotely and non-intrusively; independent of water turbidity, light level and depth^20^, offering a useful tool in conservation studies. It is possible to monitor continuously the acoustic activity of organisms living in a habitat (the biocenosis) from the specific level^21^ to the population level^22^,^23^. Data on marine soundscapes exist for many locations, including sites within the Pacific, Atlantic and Indian Oceans^19^,^24^,^25^, allowing preliminary descriptions of the variation in acoustic activity between and within environments^16^. Variations in soundscapes can be linked with habitat conditions and the communities they support^26^,^27^. Nedelec *et al*.^28^, for instance, showed that 4 acoustic parameters could provide information on the composition of living communities of coral reefs. The number of sounds produced by snapping shrimps and sound levels above 630 Hz were negatively correlated with live coral cover, density and diversity of fishes. The full band and low frequency band sound levels however were positively correlated with sea state, depth and coral type.

Although acoustic monitoring appears to provide a valuable tool for the survey of coral reefs, and more generally of the marine environment, studies have so far been restricted to snapshots. To date, no long term time series of acoustic indices related to biodiversity has been obtained in the marine environment, particularly for coral reefs^29^. Full assessment of the methodology requires confirmation that reef biodiversity is indeed correlated with acoustic data. Recent attempts to apply acoustic-based indices to the marine environment have been made^30^,^31^,^32^,^33^ and the use of such metrics as tools for monitoring marine habitats appears promising. In the present study, the potential of soundscape analysis for the monitoring of coral reefs status was tested. Passive acoustics recordings were obtained from the coral reef at Moorea Island, French Polynesia in order to examine variations in two acoustic indices, i.e. the sound pressure level and the acoustic complexity index (ACI, which quantifies soundscape complexity by computing the variability of amplitudes in sound recordings^34^), in relation to fish community and benthic habitats both inside and outside marine protected areas located on the north coast of Moorea Island.

## Results

### Characterisation of the study sites

**Figure 1:**
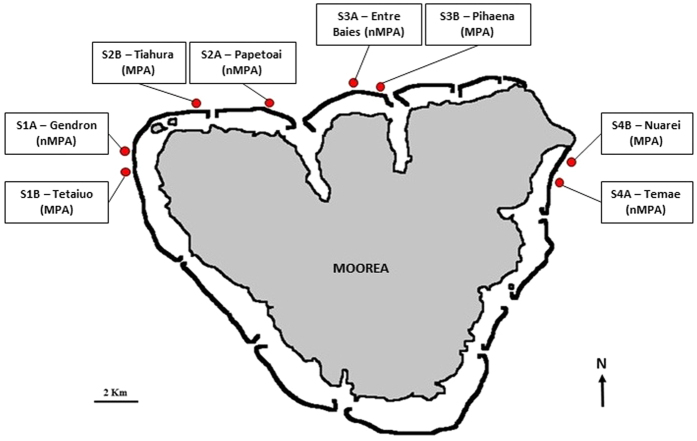
Map showing the localization of the 8 study sites on the north coast of Moorea Island. Map drawn by the authors from an aerial photograph of Moorea taken by the CRIOBE in 2008 from a private plane.

The habitat substrate and fish communities were surveyed during visual observations performed during day-time at 8 different sites, i.e. couplets of 4 Marine Protected Areas (MPAs) and 4 non Marine Protected Areas (nMPAs) located nearby (Table 1, Fig. 1). The living coral cover was significantly different between the study sites (Kruskal-Wallis, χ^2^ = 19.64, df = 7, P = 0.006). S2A and S3A presented a higher cover of live coral and S4A and S4B a lower cover. No significant differences were found between the sites for the remaining substrates, i.e. rubble, sand, pavement and/or macro-algae (Kruskal-Wallis, χ^2^ = 4.62–12.40, df = 7, P = 0.061–0.706). Significant differences between sites were also observed for the number of fish species and the number of fishes (Kruskal-Wallis, χ^2^ = 16.22, df = 7, P = 0.023 and χ^2^ = 19.40, df = 7, P = 0.007 respectively). Differences were also observed for the number of vocal fish species (Kruskal-Wallis, χ^2^ = 17.77, df = 7, P = 0.013) and the number of vocal fishes (Kruskal-Wallis, χ^2^ = 18.22, df = 7, P = 0.011). No significant differences were found between nMPAs and MPAs for any variables obtained during the visual surveys (Mann-Whitney tests, U_4, 4_ = 4–6, P > 0.05).

### Variations of ambient sound pressure level are linked to coral cover

**Figure 2:**
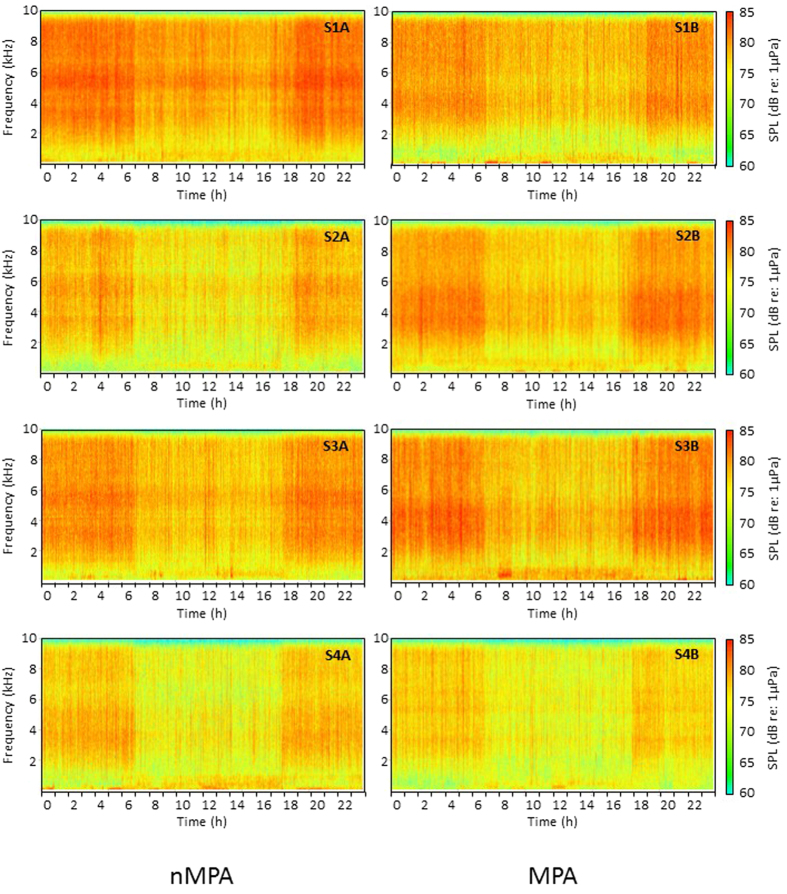
Spectrograms showing examples of 24 h of data from each recording sites. Spectrograms were obtained by concatenating 1s sound samples per hour during the first 24h of the first replicate (FFT size 512 points, Hamming window).

**Figure 3:**
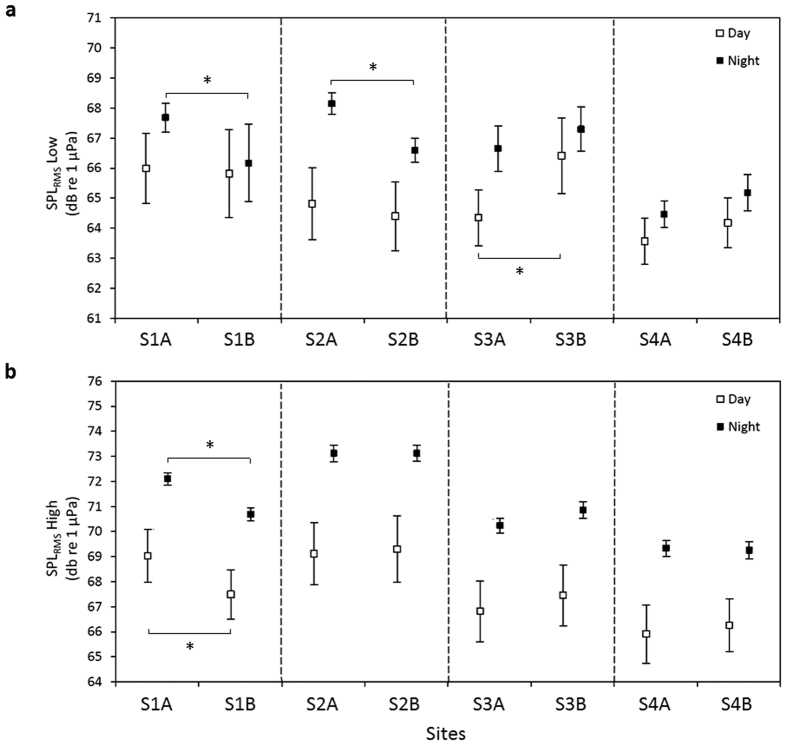
Mean ± SD values of ambient sound pressure levels (SPL_RMS_). Recorded during day and night in the low (**a**) and high (**b**) frequency bands at each site. Non-MPA/MPA pairs are separated by dashed lines. Asterisks show significant differences between nMPAs and their respective adjacent MPAs.

The average ambient sound pressure level (SPL) showed a marked diel pattern for each site, with higher SPLs recorded during the night than during the day (Fig. 2). SPLs increased at dusk (17:00–19:00) and decreased at dawn (05:00–07:00). In the low frequency band (20 Hz–2 kHz), the difference between night-time and day-time was significant for 4 sites out of 8, i.e. S1A, S2A, S2B and S3A (Kruskal-Wallis, χ^2^ = 246.75, df = 15, P < 0.001) (Fig. 3a). In the high frequency band (2–10 kHz), all sites showed significantly higher average SPLs at night (Kruskal-Wallis, χ^2^ = 537.73, df = 15, P < 0.001) (Fig. 3b). Recorded ambient SPLs showed significant differences between sites during the day (SPL_RMS_ Low: Kruskal-Wallis, χ^2^ = 16.53, df = 7, P = 0.02; SPL_RMS_ High: Kruskal-Wallis, χ^2^ = 133.39, df = 7, P < 0.001) and during the night (SPL_RMS_ Low: Kruskal-Wallis, χ^2^ = 140.09, df = 7, P < 0.001; SPL_RMS_ High: Kruskal-Wallis, χ^2^ = 177.26, df = 7, P < 0.001). Post-hoc pairwise comparisons between nMPAs and their adjacent MPAs revealed significant differences in the low frequency band between S3A/S3B during the day (Tukey test, t = 3.77, P = 0.006), and between S1A/S1B (Tukey test, t = 3.54, P = 0.01) and S2A/S2B (Tukey test, t = 3.90, P < 0.005) during the night. In the high frequency band, S1A/S1B significantly differed both during the day (Tukey test, t = 3.69, P = 0.006) and the night (Tukey test, t = 3.71, P = 0.006). Night-time SPL in the low frequency band was positively correlated with living coral cover (ρ = 0.70, P = 0.05) and negatively correlated with rubble cover (ρ = −0.82, P = 0.01). No correlation was found for SPL in the low frequency band during day-time (Table 2). SPL in the high frequency band was positively correlated with living coral cover both during the day (ρ = 0.69, P = 0.07) and the night (ρ = 0.70, P = 0.05) (Table 2).

### Variations of acoustic complexity index are linked to fish diversity

**Figure 4:**
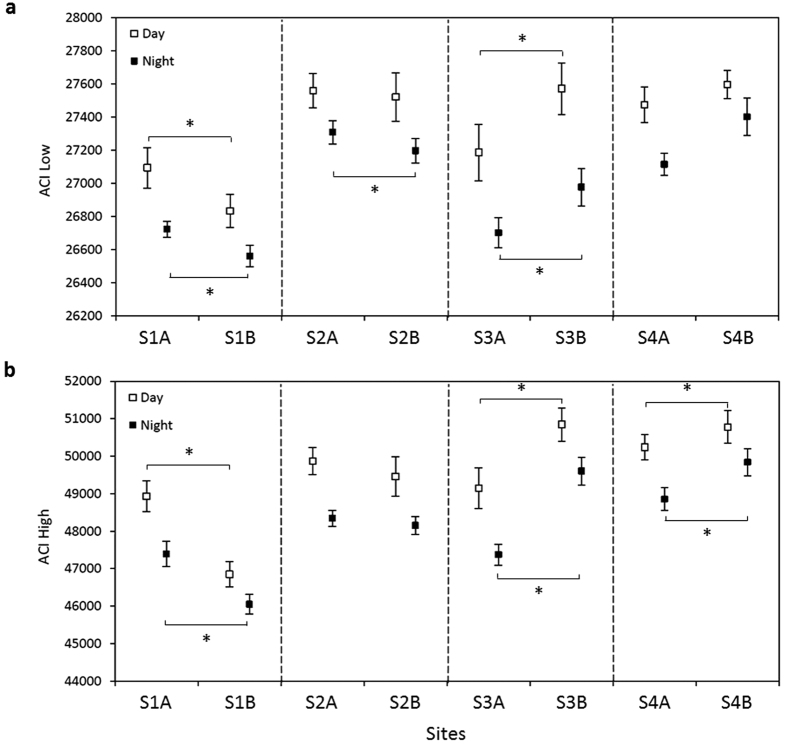
Mean ± SD values of ACI. Calculated from recordings made during day and night in the low (**a**) and high (**b**) frequency bands at each site. Non-MPA/MPA pairs are separated by dashed lines. Asterisks show significant differences between nMPAs and their respective adjacent MPAs.

Acoustic Complexity Index (ACI) values also showed a marked diel pattern for each site, with a decrease at dusk (17:00–19:00) and an increase at dawn (05:00–07:00). In the low frequency band, ACIs were significantly higher during the day at all sites (Kruskal-Wallis, χ^2^ = 738.39, df = 15, P < 0.001) (Fig. 4a), as were ACIs in the high frequency band (Kruskal-Wallis, χ^2^ = 941.09, df = 15, P < 0.001) (Fig. 4b). ACIs showed significant differences between sites during the day (ACI Low: Kruskal-Wallis, χ^2^ = 283.20, df = 7, P < 0.001; ACI High: Kruskal-Wallis, χ^2^ = 373.06, df = 7, P < 0.001) and night (ACI Low: Kruskal-Wallis, χ^2^ = 107.40, df = 7, P < 0.001; ACI High: Kruskal-Wallis, χ^2^ = 456.72, df = 7, P < 0.001). Comparisons between nMPAs and their adjacent MPAs revealed significant differences in the low frequency band between S1A/S1B (Tukey test, t = 5.58, P < 0.001) and S3A/S3B (Tukey test, t = 8.78, P < 0.001) during the day; and between S1A/S1B (Tukey test, t = 4.24, P < 0.001), S2A/S2B (Tukey test, t = 3.76, P = 0.004) and S3A/S3B (Tukey test, t = 5.50, P < 0.001) during the night. In the high frequency band, except for S2A/S2B, all nMPAs and their adjacent MPAs were significantly different both during the day (Tukey test, t = 4.64–15.23, P < 0.001) and the night (Tukey test, t = 8.85–17.45, P < 0.001). During day and night-times respectively, ACI values in the low frequency band tended to be and were positively correlated with the number of fish species observed during day-time surveys (day: ρ = 0.67, P = 0.07 ; night: ρ = 0.81, P = 0.01). Likewise, ACI values in the low frequency band were positively correlated with vocal fish species counted during day-time surveys (day: ρ = 0.71, P = 0.04 ; night: ρ = 0.78, P = 0.03) (Table 2). The two sites with the highest number of vocal fishes were MPAs (S2B and S4B) and these sites showed higher ACI values in the low frequencies (although this was not significant). ACI values of day-time recordings were positively correlated with the index of biodiversity H in the low frequency band (ρ = 0.76, P = 0.03) and tended to be positively correlated with H in the high frequency band (ρ = 0.67, P = 0.07). No correlations were found for night-time recordings (Table 2). Interestingly, the two sites presenting highest biodiversity were MPAs (S3B and S4B) and they showed significantly higher ACI values than the nMPA site presenting highest biodiversity (S3A) (Tukey test, t = 13.01–13.20, P < 0.001).

## Discussion

Climate change put coral reefs under several concomitant threats. It is critical to develop new tools to quickly measure the degradation rate and/or the recovery capacity of these hot spots of biodiversity. Passive acoustics recording demonstrated that two indices can be used to compare the characteristics of coral reefs in Moorea, French Polynesia. First, the average sound pressure level of ambient sound was correlated with the percentage of living coral covering the substratum (Table 2). Second, the complexity of the recordings positively correlated with the fish species diversity and the number of vocal fish species (Table 2). Even though performed on a single island during the warm season, these results encourage the use of acoustic indices to be repeated on other reefs and for longer time periods.

Sound pressure levels were positively correlated with coral cover, i.e. the more coral covering the substrate, the noisier the environment. Numerous studies have reported higher sound levels in denser terrestrial habitats. For example, habitats with a greater canopy cover, i.e. forest and woodland, were characterized by higher avian sound levels than savanna and shrub land^35^. Likewise, old growth forest produced a higher level of biophony than open grassland^7^. As higher coral cover may result in larger fish assemblages^6^, we may assume increased sound levels in the low frequency band actually reflect fish density, as many species produce sounds at dusk and during the night^18^,^36^. The higher levels in the high frequency band may reflect a higher abundance of snapping shrimp and other crustaceans, producing higher pitched sound during day and night and relying on corals for shelter and/or nutrition^37^. A recent study showed that reefs in good condition produce ambient sounds at higher sound levels, which will consequently propagate further than sounds of degraded reefs^38^. The positive correlation between sound level and coral cover found in this study confirms that reefs with higher coral cover are noisier and more attractive to fish. This result is confirmed by the negative correlation found between sound level in the low frequency band and the rubble cover suggesting a lower vocal activity at more degraded sites. Monitoring sound levels at reefs therefore allows comparisons of their coral cover and their characteristics to be made. Acoustical differences in the low frequency band may provide information on the different fish communities and assemblages. Further investigations should help to identify the different vocal species calling at night and to define the acoustic communities. By doing so, sentinel species that might be abundant in coral-dominated habitats might be identified. As an example, a study conducted in a Mediterranean reserve reported that the presence of the brown meagre (*Sciaena umbra*), an easily detectable vocal species, was associated with a cluster of 16 other fishes^39^ making *S. umbra* a good indicator of fish diversity in the milieu.

Acoustic indices can provide a quick appraisal of the diversity of calling species and permit a comparison of soundscapes^9^. Among these indices, acoustic richness index and acoustic dissimilarity index for instance, both appeared to be correlated with the number of acoustic species present in the community. They show lower values in a degraded dry lowland coastal forest^10^. Among these, acoustic complexity index was used to estimate avian community based on the spectral complexity of the soundscapes^34^,^40^ and evaluate vegetation structure^40^,^41^. More recently, the ACI was applied to recordings performed in freshwater ponds where it positively correlated with the richness and abundance of underwater sound types^42^. In Moorea, ACI in the low frequency band correlated positively with the number of fish species and with the number of vocal species (during day and night), while ACI in both low and high frequency bands correlated positively with the Shannon-Wiener index of fish diversity only during the day. Previous applications of ACI to marine environments have not always resulted in correlating fish species assemblages with soundscape complexity^30^,^32^. The current result confirms that ACI would be suitable index for evaluating biophony and species richness within marine soundscapes as recently shown by Harris *et al*.^33^ who identified ACI as a robust index for use on temperate reefs. In the present study, the application of ACI particularly allowed comparisons to be made between adjacent non protected and protected sites. Thus, two MPAs, i.e. Pihaena (S3B) and Nuarei (S4B), displayed higher acoustic complexity than their respective nMPAs located nearby, i.e. Entre Baies (S3A) and Temae (S4A). For the S1A/S1B pair however, the nMPA (S1A - Gendron) displayed higher H index and hence significantly higher ACI values than its adjacent MPA (S1B - Tetaiuo). Finally, S2A (Papetoai - nMPA) and S2B (Tiahura - MPA) showed similar H indices and no significant difference between ACI values was observed. Marine protected areas provide a worldwide strategy for the conservation of marine biodiversity. MPAs apply restrictions to fisheries and to the anchorage of vessels, for instance, with the aim of providing safer places for marine organisms. More fish species are present at such sites, with higher fish densities and higher biodiversity indices than non-protected sites. In December 2015, the United Nations conference on climate change, COP21, held in Paris, France, succeeded in reaching a universal agreement on climate and particularly agreed to keep expanding the existing coverage of terrestrial, coastal and marine protected areas and increase the number of MPAs from 3% of the world’s marine and coastal zones up to, at least, 10% by 2020. To our knowledge, the present study is the first one to demonstrate that acoustic monitoring can play an important role in the future evaluation of the efficacy and appropriateness of marine protected areas and how they are designated and managed. The monitoring of soundscapes would also permit a rapid assessment of disturbances triggered by acute natural perturbations. As an example, Indeck *et al*.^43^ showed that the proliferation of a toxic micro algae caused a significant decrease in ambient noise level due to its lethal effects on fishes and shrimps. Coral reefs face similar threats including cyclones (e.g. cyclone Oli hit the western coast of Moorea in 2010) and crown-of-thorns starfish (COTS) outbreaks (the last event in Moorea occurred during the last decade)^44^.

To conclude, bioacoustics has only recently been applied to questions related to higher ecological levels such as communities or ecosystems. This study demonstrates that ambient sound level and acoustic complexity can be used to appraise the percentage of coral cover and fish diversity in coral reefs. Future investigations should confirm these results, and build on the observation that a more complex soundscape may be associated with higher fish diversity in marine protected areas. By doing so, acoustics will provide reliable proxies to be included in conservation plans in order to judge the efficacy of marine protected areas. Moreover, acoustic monitoring may also contribute to understanding how environmental changes may affect marine animals, in their distribution pattern or migratory behaviour for instance. Examining how environmental change translates into soundscape alteration appears to be a promising field of study.

## Methods

### Study sites

The study was carried out during the warm season, from March to June 2015, on the north coast of Moorea Island (French Polynesia–17°30′S, 149°5′W). Study sites were composed of couplets made of 4 non-Marine Protected Areas (nMPAs): Gendron (S1A), Papetoai (S2A), Entre Baies (S3A) and Temae (S4A); associated with 4 Marine Protected Areas (MPAs) located nearby: Tetaiuo (S1B), Tiahura (S2B), Pihaena (S3B), Nuarei (S4B) respectively^45^,^46^.

### Biological data collection

At each site, three 20 m transect lines were placed parallel to the shore, 2.5 m apart. Habitat and fish surveys were performed in March 2015. For habitat surveys, living coral-macro algae-pavement-sand or rubble substrate was recorded every metre using the line intercept transect method, defining the percentage of cover for the different substrates along the transect. Fish surveys were conducted along the same transects as the substrate surveys with a width of 1 m. Active (not hiding in coral) juvenile and adult fishes were recorded to species level. The same diver conducted fish surveys during two passes over each transect^6^. On the first pass, the diver swam quickly to record mobile fishes that swam within transects but usually fled with the divers’ approach. On the second pass, the diver swam more slowly to record site-attached species. All surveys were carried out during the day-time (06:00 to 17:00), as fish could not be identified accurately at night because of poor visual conditions. Survey data were used to calculate the number of fish species, the number of fishes and the Shannon-Wiener index of diversity (H) for the 8 sites. Based on the list of species obtained, the number of “known-to-be” vocal species and number of vocal fishes were also calculated.

### Acoustic data collection and processing

Two autonomous underwater digital spectrogram (DSG) long-term acoustic recorders (Loggerhead Instruments, Sarasota, FL, USA) were deployed. Each was connected to a HTI96-min hydrophone (sensitivity: −180 dB re: 1 V for a sound pressure of 1 μPa; High Tech Inc, Long Beach, MS, USA). The recorders were scheduled to record sounds for 5 min every hour for 48 hours at a sampling rate of 48 kHz (16-bit resolution). Recorders were attached to blocks of lead, positioned on the outer slope of the reef at 10 m depth by professional divers and remained in place during the course of each replicate. Simultaneous recordings were made at two sites, i.e. an nMPA and its nearby MPA. Sites were therefore coupled as follows: S1A/S1B, S2A/S2B, S3A/S3B and S4A/S4B. For each site, 3 replicates were obtained with a 3-week time interval between them, and were distributed on different lunar cycle stages. The positions of the recorders were localized with a GPS for each replicate. Replicates made at one site were no more than 10 m apart. At the end of each replicate sampling period, data were retrieved. Recordings were downloaded at a sampling rate of 20 kHz, providing an analysis range of 1 Hz to 10 kHz. A 20 Hz high-pass filter was applied to all recordings to eliminate very low frequencies. The root mean square (RMS) of the Sound Pressure Level (SPL, in dB re: 1 μPa) was measured over a low frequency band (20 Hz to 2 kHz) (SPL_RMS_ Low) and also over a high frequency band (2 kHz to 10 kHz) (SPL_RMS_ High) for each sample using Avisoft SASLab Pro 5.2.07 software (Avisoft Bioacoustics, Glienicke, Germany). The acoustic spectrum was partitioned into these 2 non-overlapping frequency bands in order to focus on the dominant sound sources within each band. The low frequency band (20 Hz to 2 kHz) corresponds to the range in which most fish species vocalize^47^ and hear^48^. This band may also include noise generated by the wind and waves. The high frequency band (2 to 10 kHz) encompassed the range that is typically dominated by snapping shrimp^14^. Boat noise covers a large frequency band and may interfere with both bands.

To examine the complexity of the soundscape, the Acoustic Complexity Index (ACI)^34^ was calculated using R and the Seewave package^49^. ACI calculates the differences in amplitude of adjacent time samples in each frequency bin within the entire signal, then sums the differences across all bins, to provide a single measure of the changing composition of the soundscape^34^. Higher values are generated by greater variability in sound level resulting from multiple sound sources or different sound types, whereas sounds with a more constant sound level produce low values. We applied this index to the low frequency (ACI Low) and high frequency bands (ACI High) (time window = 0.5 s, Fast Fourier Transform FFT, 512 points, Hamming window, matching a 39.06 Hz resolution) over the entire length of the recordings.

Even though boat traffic at study sites was low and hydrophones were removed prior to storms, recordings showing an unusually elevated SPL or extreme ACI values (e.g. due to boat noise, animals probing the recording device or during rain episodes) were detected and removed from the analysis. Detailed weather conditions including sea state, wind speed and direction, temperature and rain can be found as Supplementary Table S1.

### Data analysis

Due to the low number of replicates, data from visual surveys were compared between sites by means of Kruskal-Wallis tests. Data obtained from visual survey in nMPAs and MPAs were compared by means of Mann-Whitney U tests. Time series of acoustic data were obtained for each site by plotting the mean values of sound pressure level (SPL_RMS_) and ACI over a 24 h period with a 1-h resolution. Time series were divided in day-time (06:00–17:00, corresponding to the time period of visual surveys) and night-time (18:00–05:00). Dawn (05:00–07:00) and dusk (17:00–19:00) were included in day-time and night-time respectively. Since data were not normally distributed (Shapiro-Wilks tests, W = 0.95–0.99, P = 0.03–0.001), between sites differences of day and night values of SPL_RMS_ and ACI were examined by means of Kruskal-Wallis tests, followed by Tukey’s post-hoc tests for pairwise comparisons. Acoustic data obtained during day-time were correlated with biological data, i.e. total number of fish species, total number of fishes, Shannon-Wiener index of diversity (H), number of vocal species, number of vocal fishes and the percentage of substrate covered with living coral, rubble, sand, pavement and macro-algae by means of Spearman’s rank correlation tests. Acoustic data obtained during night-time were also correlated with biological data. Even though fish survey were only performed during day-time, fishes preferentially sing at night, and correlation with night values could provide important information too. All analyses were two-tailed, at α = 0.05 and carried out in R 3.1.2 (R Core Team, 2014) using customized scripts.

## Additional Information

**How to cite this article**: Bertucci, F. *et al*. Acoustic indices provide information on the status of coral reefs: an example from Moorea Island in the South Pacific. *Sci. Rep.* **6**, 33326; doi: 10.1038/srep33326 (2016).

## Supplementary Material

Supplementary Information

## Figures and Tables

**Table 1:** Results of the visual surveys performed at the 8 sites of the north coast of Moorea.

Site	Type	Number of species	Number of fishes	H	Number of vocal species	Number of vocal fishes	Living coral cover %	Rubble %	Pavement %	Sand %	Macro-algae %
S1A	nMPA	35 ± 2	278 ± 32	2.82	20 ± 3	185 ± 28	37 ± 12	5 ± 3	53 ± 6	1 ± 2	4 ± 5
S1B	MPA	40 ± 2	417 ± 118	2.42	23 ± 3	349 ± 123	33 ± 2	14 ± 12	50 ± 16	1 ± 1	2
S2A	nMPA	44 ± 7	342 ± 98	2.88	29 ± 6	252 ± 84	54 ± 9	5 ± 7	32 ± 12	4 ± 3	5 ± 3
S2B	MPA	55 ± 5	623 ± 177	2.90	36 ± 4	533 ± 167	43 ± 4	8 ± 11	48 ± 14	0	1 ± 1
S3A	nMPA	34 ± 7	187 ± 135	3.17	21 ± 3	140 ± 119	50 ± 5	3 ± 4	43 ± 10	1 ± 1	3 ± 1
S3B	MPA	33 ± 7	140 ± 4	3.34	22 ± 5	109 ± 13	31	6 ± 10	60 ± 9	1 ± 1	2 ± 5
S4A	nMPA	46 ± 5	485 ± 94	2.75	30 ± 4	442 ± 95	15 ± 1	20 ± 4	64 ± 6	1 ± 1	0
S4B	MPA	51 ± 4	1055 ± 12	3.28	34 ± 3	295 ± 22	11 ± 5	31 ± 7	57 ± 5	1 ± 1	0

Values are mean ± SD.

**Table 2:** Results of Spearman’s rank correlation tests performed between acoustic and biological data.

		Time	Number of species	Number of fishes	H	Number of vocal species	Number of vocal fishes	Living coral %	Rubble %	Pavement %	Sand %	Macro-algae %
SPL_RMS_	Low	Day	ρ = −0.48; P = 0.23	ρ = −0.52; P = 0.19	ρ = 0.07; P = 0.88	ρ = −0.52; P = 0.19	ρ = −0.52; P = 0.19	ρ = 0.24; P = 0.58	ρ = −0.43; P = 0.29	ρ = −0.09; P = 0.84	ρ = 0.30; P = 0.47	ρ = 0.53; P = 0.17
		Night	ρ = −0.39; P = 0.33	ρ = −0.62; P = 0.12	ρ = 0.19; P = 0.66	ρ = −0.47; P = 0.24	ρ = −0.62; P = 0.11	**ρ = 0.70; P = 0.05**	**ρ = −0.82; P = 0.01**	ρ = −0.55; P = 0.17	ρ = 0.62; P = 0.10	ρ = 0.28; P = 0.47
	High	Day	ρ = 0.07; P = 0.86	ρ = 0.12; P = 0.79	ρ = −0.12; P = 0.79	ρ = 0; P = 1	ρ = 0.12; P = 0.79	**ρ = 0.69; P = 0.07**	ρ = −0.42; P = 0.30	ρ = −0.63; P = 0.10	ρ = 0.11; P = 0.80	ρ = 0.51; P = 0.20
		Night	ρ = 0.06; P = 0.89	ρ = −0.05; P = 0.93	ρ = −0.07; P = 0.88	ρ = 0; P = 1	ρ = 0.05; P = 0.93	**ρ = 0.70; P = 0.05**	ρ = −0.49; P = 0.22	ρ = −0.52; P = 0.19	ρ = 0.11; P = 0.80	ρ = 0.53; P = 0.17
ACI	Low	Day	**ρ = 0.67; P = 0.07**	ρ = −0.12; P = 0.79	**ρ = 0.76; P = 0.03**	**ρ = 0.71; P = 0.04**	ρ = −0.12; P = 0.79	ρ = −0.28; P = 0.50	ρ = 0.29; P = 0.49	ρ = 0.19; P = 0.66	ρ = −0.08; P = 0.85	ρ = −0.35; P = 0.40
		Night	**ρ = 0.81; P = 0.01**	ρ = 0.28; P = 0.50	ρ = 0.38; P = 0.36	**ρ = 0.78; P = 0.03**	ρ = 0.28; P = 0.50	ρ = −0.17; P = 0.70	ρ = 0.38; P = 0.35	ρ = 0.05; P = 0.93	ρ = 0.03; P = 0.95	ρ = −0.36; P = 0.38
	High	Day	ρ = 0.54; P = 0.17	ρ = −0.17; P = 0.70	**ρ = 0.67; P = 0.07**	ρ = 0.55; P = 0.17	ρ = −0.17; P = 0.70	ρ = −0.47; P = 0.24	ρ = 0.35; P = 0.40	ρ = 0.50; P = 0.22	ρ = −0.11; P = 0.80	ρ = −0.43; P = 0.28
		Night	ρ = 0.58; P = 0.13	ρ = −0.05; P = 0.93	ρ = 0.57; P = 0.15	ρ = 0.59; P = 0.13	ρ = −0.04; P = 0.93	ρ = −0.57; P = 0.15	ρ = 0.48; P = 0.23	ρ = 0.55; P = 0.17	ρ = −0.03; P = 0.95	ρ = −0.52; P = 0.19

Significant correlations at α = 0.05 and tendencies are displayed in bold.
