# Supplementary material for: Acoustic indices provide information on the status of coral reefs: an example from Moorea Island in the South Pacific
Source: Sci Rep. 2016 Sep 15;6:33326. doi: 10.1038/srep33326 (PMC5024098; doi:10.1038/srep33326)
Supplement: Supplementary Information [file srep33326-s1.pdf]

Acoustic indices provide information on the status of coral reefs: an example from Moorea Island in the South Pacific

Frédéric Bertucci, Eric Parmentier, Gaël Lecellier, Anthony D. Hawkins, David Lecchini

Supplementary Table S1. Detailed weather conditions recorded on the north coast of Moorea during the 3 replicates. Colors refer to 48h recording periods of the 4 couplets made of non-Marine Protected Areas (nMPAs) and Marine Protected Areas (MPAs): yellow = S1A/S1B, orange = S2A/S2B, green = S3A/S3B and blue = S4A/S4B. The second replicate of S4A/S4B lasted 72h due to technical issues but only the first 48h were considered for analysis. Data are from www.windguru.cz archives.

1	Wind speed (knots)				Wind direction				Wave (m)				Wave direction				Temperature (°C)				Rain (mm/3h)			
Date / Time	02	08	14	20	02	08	14	20	02	08	14	20	02	08	14	20	02	08	14	20	02	08	14	20
01.03.2015	11	7	10	10	↙	←	↙	↙	1.5	1.4	1.4	1.3	↗	↗	→	→	28	28	28	28				2.7
02.03.2015	12	12	13	12	↙	↙	↓	↓	1.3	1.3	1.3	1.3	↗	↗	↗	↗	28	28	28	28				0.6
03.03.2015	13	14	16	15	↓	↓	↓	↙	1.2	1.2	1.3	1.4	↗	↗	↗	↗	28	28	28	28	5.2			
04.03.2015	13	12	14	16	↙	↙	↙	↙	1.4	1.3	1.4	1.5	↗	↗	↗	↗	28	28	28	28			2	0.6
05.03.2015	12	14	15	13	↙	↙	↙	↙	1.4	1.4	1.5	1.5	↗	↗	↗	↖	28	28	28	28		0.5		
06.03.2015	13	13	14	12	←	←	↙	↙	1.5	1.4	1.4	1.4	↗	↗	↑	↗	28	28	28	28				
07.03.2015	12	10	9	8	↙	↙	↙	↓	1.3	1.2	1.2	1.2	↗	↗	↑	↑	28	28	28	28				1.1
08.03.2015	9	6	6	3	↓	↓			1.1	1.1	1.1	1.2	↑	↖	↑	↑	28	28	28	28				
09.03.2015	5	6	6	3		↙	↙		1.4	1.4	1.4	1.3	↑	↑	↑	↑	27	27	28	28				
10.03.2015	3	6	8	7			↙	↙	1.3	1.3	1.2	1.2	↖	↖	↖	↖	28	28	28	28				
11.03.2015	7	8	7	7	↙	↙	↙	↙	1.2	1.2	1.2	1.2	↖	↖	↖	↖	28	28	28	29				
12.03.2015	8	8	8	8	↙	↙	↙	↓	1.2	1.2	1.2	1.2	↖	↑	↖	↗	28	28	28	29				
13.03.2015	7	10	14	8	↙	↓	↘	↘	1.2	1.3	1.4	1.5	↗	↗	↗	↗	28	28	28	29		1.8	2.4	
14.03.2015	10	14	16	21	↘	↘	↘	↓	1.5	1.6	1.8	1.8	↗	↗	↗	↗	28	28	28	27	0.6	0.4	2.9	8.4
15.03.2015	29	24	15	11	↘	↘	→	↗	2.5	2.9	2.5	2	→	↘	→	→	28	28	29	28	17	6.7		
16.03.2015	9	9	8	8	↗	↗	↑	↑	1.9	1.8	1.7	1.6	→	→	→	↘	28	28	28	28				
17.03.2015	5	4	5	9				↖	1.6	1.5	1.5	1.4	↘	↘	↘	↘	27	27	27	28				
18.03.2015	14	15	15	15	↖	↖	↖	↖	1.5	1.6	1.8	1.9	↘	↘	↘	↘	28	28	28	28				2.3
19.03.2015	18	26	29	26	↖	↙	↙	↙	2	2.1	2.8	3	↘	↘	↙	↙	28	28	28	28		2.2		0.6
20.03.2015	22	18	17	19	↙	↙	↙	↙	2.7	2.5	2.5	2.7	↘	→	↗	↗	28	28	28	28				

2	Wind speed (knots)				Wind direction				Wave (m)				Wave direction				Temperature (°C)				Rain (mm/3h)			
Date / Time	02	08	14	20	02	08	14	20	02	08	14	20	02	08	14	20	02	08	14	20	02	08	14	20
29.03.2015	9	8	9	8	↓	↓	↓	↙	1.3	1.2	1.2	1.1	↗	↗	↗	↗	28	29	28	28				
30.03.2015	8	11	12	12	↙	↙	↙	↙	1.1	1.2	1.4	1.7	↗	↗	↗	↗	28	28	28	28				
31.03.2015	13	12	12	10	↙	↙	↓	↙	1.9	2	1.9	1.8	↗	↗	↗	↗	28	28	28	28		0.3		
01.04.2015	9	8	7	7	↙	←	←	←	1.7	1.6		1.5	↗	↗		↗	28	28	28	28				
02.04.2015	10	9	10	8	←	↙	↓	↓	1.5	1.5	1.5	1.5	↗	↗	↗	↗	28	28	28	28				
03.04.2015	4	6	7	10			←	←	1.5	1.5	1.4	1.4	↗	↗	↑	↑	28	28	28	28				
04.04.2015	12	11	10	12	←	←	←	↖		1.4	1.4	1.5		↑	↑	↗	28	28	28	28				
05.04.2015	16	15	15	15	↖	←	↖	↖	1.8	1.9	2	2	↗	↗	↗	↗	28	28	28	28			1.8	0.9
06.04.2015	15	9	9	12	↖	←	↙	↙	2	1.9	1.8	1.8	↗	↗	↗	↗	28	28	28	28	0.6	0.7	0.4	1
07.04.2015	12	14	11	7	↙	↙	↓	↓	1.7	1.7	1.6	1.5	↗	↗	↗	↗	28	28	28	28				
08.04.2015	7	9	6	5	↙	↙	↙		1.5	1.5	1.4	1.3	↗	↗	↗	↗	28	28	28	28				
09.04.2015	5	6	6	6				↖	1.3	1.2	1.2	1.2	↗	↗	↗	↗	28	28	28	28	0.4			
10.04.2015	10	10	9	9	←	↖	←	↖	1.2	1.2	1.2	1.2	→	→	→	→	28	28	28	29	3.2	0.3		
11.04.2015	11	8	9	6	↖	←	←		1.3	1.3	1.3	1.3	↗	↗	↗	↗	28	28	28	28	1	0.4	0.7	1

3	Wind speed (knots)				Wind direction				Wave (m)				Wave direction				Temperature (°C)				Rain (mm/3h)			
Date / Time	02	08	14	20	02	08	14	20	02	08	14	20	02	08	14	20	02	08	14	20	02	08	14	20
20.04.2015	18	19	17	15	←	←	↙	↙	2.1	2.2	2.1	2	↗	↗	↑	↑	28	28	28	28	0.3	1.8		
21.04.2015	15	17	16	17	↙	↙	↙	↙	2	2	2.1	2.1	↑	↑	↑	↑	28	28	28	28				
22.04.2015	17	15	13	13	←	↙	↙	↙	2.1	2	1.9	1.9	↑	↑	↑	↑	28	27	28	28				
23.04.2015	14	15	14	14	↙	↙	↙	↙	1.8	1.8	1.7	1.6	↑	↖	↗	↗	28	28	28	28	0.4			
24.04.2015	13	11	13	14	↙	←	←	←	1.5	1.4	1.4	1.4	↗	↗	↗	↗	28	28	28	28				
25.04.2015	15	15	13	16	←	←	←	↖	1.5	1.5	1.5	1.6	↗	↗	↗	↗	28	27	27	28				
26.04.2015	16	15	15	14	↖	←	←	↙	1.6	1.7	1.8	1.7	↗	↗	↗	↗	28	28	28	28		1.3		7.7
27.04.2015	9	6	5	7	↙	↓		↓	1.6	1.6	1.7	1.7	↗	↗	↗	↗	28	28	28	28				
28.04.2015	6	1	2	7				↖	1.7	1.6	1.6	1.6	↗	↗	↗	↗	28	28	28	28				
29.04.2015	10	9	8	9	↖	↖	↖	↖	1.6	1.7	1.7	1.6	↗	↑	↑	↑	28	28	28	28	2.5	0.7		
30.04.2015	11	10	12	13	←	↙	↙	←	1.5	1.5	1.4	1.4	↗	↗	↗	↗	28	28	28	28				
01.05.2015	14	16	14	14	↙	↙	↙	←	1.4	1.5	1.5	1.5	↗	↗	↗	↗	28	28	28	28		2.8		0.9
02.05.2015	16	15	15	16	←	←	←	↙	1.5	1.4	1.7	2.1	↗	↗	↗	↗	28	28	28	28			0.6	4.4
03.05.2015	15	14	13	11	←	↙	↙	←	2.5	2.6	2.5	2.3	↗	↗	↗	↗	28	27	28	28	4.1	0.8		3.1
04.05.2015	11	11	11	11	↙	↙	↙	↙	2.2	2.2	2.2	2.2	↗	↗	↗	↗	28	28	28	28	1.4	0.5		
05.05.2015	11	11	9	3	↙	↓	↓		2.2	2.2	2.1	2.1	↗	↗	↗	↗	28	28	28	28	1.4	0.9	0.9	9.8
06.05.2015	7	4	3	5	←				2.2	2.1	2.1	2.1	↗	↗	↗	↗	28	28	28	28				
07.05.2015	9	9	9	6	←	↙	↙		2	2	2	2.1	↗	↗	↗	↑	27	27	28	27	1.1	5.7		3
08.05.2015	6	5	9	11	↘		↘	↘	2.2	2.2	2.1	2.1	↑	↑	↑	↑	27	27	27	28	1.1		1.9	1.4
